# Mechanisms of Neuroprotection by Protein Disulphide Isomerase in Amyotrophic Lateral Sclerosis

**DOI:** 10.1155/2011/317340

**Published:** 2011-04-11

**Authors:** Adam K. Walker, Julie D. Atkin

**Affiliations:** ^1^Department of Biochemistry, La Trobe Institute for Molecular Science, La Trobe University, Bundoora, VIC 3086, Australia; ^2^Howard Florey Institute, Florey Neuroscience Institutes and Centre for Neuroscience, The University of Melbourne, Parkville, VIC 3010, Australia

## Abstract

Amyotrophic lateral sclerosis (ALS) is a devastating neurodegenerative disease characterised by the progressive loss of motor neurons, leading to paralysis and death within several years of onset. Although protein misfolding is a key feature of ALS, the upstream triggers of disease remain elusive. Recently, endoplasmic reticulum (ER) stress was identified as an early and central feature in ALS disease models as well as in human patient tissues, indicating that ER stress could be an important process in disease pathogenesis. One important chaperone induced by ER stress is protein disulphide isomerase (PDI), which is both upregulated and posttranslationally inhibited by S-nitrosylation in ALS. In this paper, we present evidence from studies of genetics, model organisms, and patient tissues which indicate an active role for PDI and ER stress in ALS disease processes.

## 1. Introduction

Amyotrophic lateral sclerosis (ALS) is a fatal, rapidly progressing neurodegenerative disorder primarily affecting motor neurons of the spinal cord, brainstem, and cortex [1]. The disease is characterised by muscle weakness leading to paralysis and death usually within several years of diagnosis [2]. Similar to other neurodegenerative diseases, protein misfolding and abnormal intracellular protein inclusions are pathological hallmarks of ALS. However, the pathogenic mechanisms of disease are yet to be elucidated [3]. Approximately 10% of ALS cases are inherited, and up to 20% of these familial cases are caused by mutations in the gene encoding superoxide dismutase 1 (SOD1) [4, 5]. SOD1-linked disease remains the most studied and best characterised form of ALS, although successful therapeutic interventions from mutant SOD1 models have thus far failed to translate to patients [1]. Additional factors involved in disease have recently been identified, including the proteins TAR DNA binding protein 43 (TDP-43) and fused in sarcoma (FUS) as key players in disease pathogenesis [6], and understanding the molecular mechanisms of ALS will allow the design of effective therapeutics in the future. Recent evidence has identified endoplasmic reticulum (ER) stress and in particular the chaperone protein disulphide isomerase (PDI), as important in ALS disease processes [7, 8]. The role of PDI as a protective factor in ALS, with a particular focus on disulphide bond-mediated SOD1 misfolding, forms the central topic of this paper.

## 2. Endoplasmic Reticulum Stress

The ER is an important organelle for the folding and posttranslational modification of many proteins, and it also acts as a significant intracellular calcium store [9]. The ER forms close associations with other organelles, including mitochondria, the Golgi apparatus and the nucleus, and is therefore a central player in cell physiology [10, 11]. Since there is a high rate of protein production in the ER, prevention of protein misfolding is an important function of this organelle. The protein folding capacity of the ER is adjusted according to requirements in order to maintain homeostasis [12].

Proteins misfold as part of normal physiology, and when the rate of protein synthesis is increased, the burden of unfolded proteins in the ER also increases. This results in ER stress, which triggers signalling pathways collectively known as the unfolded protein response (UPR) [13]. Cellular insults including dysregulation of calcium stores, changes in the ER redox state, nutrient deprivation as well as disturbances in the Golgi, endosomal and vesicular transport systems, and induction of mitochondrial-associated apoptosis, can all elicit ER stress [14–16].

The UPR is a homeostatic mechanism which is initially protective by upregulation of chaperone proteins such as PDI, inhibition of general protein translation, and expansion of ER volume. The UPR is mediated by activation of three upstream sensors of ER stress, namely, PKR-like endoplasmic reticulum kinase (PERK), inositol-requiring kinase 1 (IRE1), and activating transcription factor 6 (ATF6) [17–21]. In the basal state, immunoglobulin binding protein (BiP) is bound to the upstream sensors, which maintains them in an inactive state. Upon ER stress induction, BiP dissociates from PERK, IRE1 and ATF6 to preferentially bind hydrophobic regions of the accumulating misfolded proteins, thereby activating the upstream sensors [13]. PERK activation leads to phosphorylation of eukaryotic translation initiation factor 2 subunit *α* (eIF2*α*), which attenuates general translation initiation, as well as upregulation of ER stress-specific proteins including activating transcription factor 4 (ATF4) [12]. IRE1 activation allows splicing of the mRNA of X box-binding protein 1 (XBP1), which is another ER stress-specific transcription factor, and activates the c-Jun N-terminal kinase (JNK) pathway, which leads to ER volume expansion [22, 23]. ATF6 is transported to the Golgi apparatus following release from BiP, where it is specifically cleaved to produce an additional ER stress-specific transcription factor [9, 22].

Prolonged ER stress, as occurs in ALS, causes cell death via apoptotic signalling. ER stress-activated apoptosis is mediated in part by the release of Ca^2+^ from the ER and upregulation of proapoptotic factors, including CCAAT/enhancer binding protein-(C/EBP-) homologous protein (CHOP), as well as activation of ER stress-specific caspases [24, 25].

## 3. ER Stress in ALS

ER stress is induced in many diseases, such as diabetes, tumour development, hypercholesterolemia and autoimmune disorders, and by viral infection [26]. Many neuronal disorders also have ER stress as a component, including Huntington's disease [27, 28], HIV-associated dementia [29], Alzheimer's disease [30], Parkinson's disease [31, 32], Creutzfeldt-Jacob disease [33] and Pick's disease [34]. In addition, aging inhibits the early protective responses of ER stress [35].

In ALS, PDI is one of the most upregulated proteins in the spinal cords of presymptomatic transgenic SOD1^G93A^ rats and mice, which are the most commonly used animal models of disease [7, 36]. Additionally, activation of the full UPR occurs prior to symptom onset in these animals, indicating an active role for ER stress in pathogenesis [7]. Indeed, ER stress occurs specifically in the vulnerable fast-fatiguable motor neurons prior to activation of other disease-associated mechanisms [37]. ER stress also occurs in sporadic ALS patient spinal cord tissues, indicating that these findings are not confined to mutant SOD1 models of disease [38, 39].

Recent genetic studies have confirmed the importance of ER stress in ALS. Genetic ablation of Ask1, a target of IRE1, or Puma, an ER-stress-related BH3-only protein, both decrease UPR activation and slow disease in SOD1^G93A^ mice [40, 41]. Ablation of Xbp1, a key modulator of the UPR, stimulated macroautophagy in motor neurons, delaying disease onset and increasing survival of SOD1^G86R^ mice [42]. Furthermore, administration of salubrinal, a small molecule inhibitor of ER stress, to SOD1^G93A^ mice significantly delayed disease progression [37]. These studies indicate that modulation of ER stress could be potentially useful in therapy for ALS patients, although further investigation of the mechanisms of protection and design of targeted molecules is required.

## 4. Protein Disulphide Isomerase

Protein disulphide isomerase (PDI) is an important chaperone induced by ER stress which is involved in the formation, reduction and isomerisation of disulphide bonds in protein substrates. PDI is therefore an important cellular defence against protein misfolding [43]. Recently, PDI upregulation was detected in both mutant SOD1 transgenic rodent and human ALS patient spinal cord tissues, suggesting that PDI could act as a protective molecule in disease [7, 38]. PDI expression is also induced in other neurodegenerative diseases such as Parkinson's and Alzheimer's diseases [30, 31], suggesting that PDI has a broad role in neurological disorders related to protein aggregation.

## 5. The Structure and Expression of PDI

There are at least 19 members of the human PDI protein family, all of which possess at least one thioredoxin-like domain and an ER targeting sequence [44]. Full-length PDI has two thioredoxin-like domains which both contain a CGHC dicysteine active site motif (termed the a and a′ domains), two intervening homologous domains (b and b′), with an x-linker region, and a C-terminal c domain containing a KDEL ER-retention motif which is also potentially involved in Ca^2+^ binding [45]. Mutagenesis studies identified the b′ domain of PDI as the principal substrate-binding site, however other domains also contribute to the binding of proteins [46]. The CGHC motifs of the a and a′ domain active sites cooperate in forming disulphide bonds, although each motif possesses independent enzymatic activity in the full-length protein [47].

Many PDI family members have not been fully characterised in terms of structure and function, although it is becoming increasingly recognised that each member has distinct substrate specificities [48, 49]. For example, whereas PDI interacts with both glycosylated and nonglycosylated proteins, ERp57 (also known as PDIA3 or Grp58) interacts specifically with glycosylated proteins [50]. Also, while ERp57^−/−^ mice are nonviable [51], genetic ablation of either of the PDI family members AGR2/Hag2 or ERdj5 produces viable mice, with specific defects in mucin production and salivary gland function, respectively, and increased ER stress in affected tissues [52, 53]. No models of genetic PDI ablation in higher eukaryotes have been reported, although PDI is essential in yeast [54]. Due to its widespread distribution and important cellular functions, genetic ablation of PDI in mammals would also likely be lethal. However, the potential for redundancy with the other PDI family members remains undetermined, and delineation of the different roles and substrate specificities of the PDI family members is an important area for future research [48].

## 6. The Functions of PDI

PDI is primarily a disulphide bond-modulating chaperone, however PDI also facilitates ER-associated degradation of misfolded proteins [55], and is involved in retro-translocation of misfolded cholera toxin from the ER to the cytoplasm by interaction with the ER transmembrane protein Derlin-1 [56, 57]. Other PDI family members are involved in calcium homeostasis and antigen presentation [44], while PDI is important for the cellular export of some proteins, such as thyroglobulin [58]. 

 PDI protects against aggregation of misfolded proteins in several neurodegenerative diseases. PDI decreases aggregation of the Parkinson's disease-associated synphilin-1 protein in neuroblastoma cells, an activity which is dependent on the presence of the CGHC active site motifs [59]. PDI also prevents aggregation of *α*-synuclein in cell-free *in vitro* systems [60], and ERp57 prevents aggregation and subsequent neurotoxicity of prion protein in cell culture [33]. PDI colocalises with ubiquitin-positive inclusions of torsin-A in a transgenic mouse model of dystonia [61], and with neurofibrillary tangles in Alzheimer's disease patient brain tissue [62]. Furthermore, PDI prevents neuronal and cardiomyocyte cell death caused by hypoxia-ischaemia in cell culture and in rodent models, at least partly by decreasing protein misfolding [63, 64]. In contrast to these findings, PDI does not decrease the number of inclusions formed by the variant of *α*1-antitrypsin linked with liver disease [65], indicating some disease and protein-specificity of PDI protection.

## 7. Subcellular Localisation of PDI

While PDI is conventionally considered as an ER lumenal protein, PDI has also been detected in the nucleus, extracellular matrix and on the cell surface, where it modulates several different functions [8, 66–72]. Cell surface PDI facilitates infection of HeLa cells by mouse polyoma virus [67], modulates thrombus formation on the surface of platelets [68] and facilitates dengue virus infection [73]. In addition, both PDI and ERp57 interact with misfolded prion protein on the cell surface, which could be important for prion accumulation and cell-to-cell transmission [74]. Also, ER stress can lead to the leakage of PDI to the cytoplasm [75]. These findings clearly indicate that PDI is found in other cellular locations under a variety of different conditions.

## 8. The Involvement of PDI in ALS

PDI is upregulated in the spinal cords of SOD1^G93A^ transgenic mice and rats at the pre-symptomatic, symptomatic and end stages of disease [7, 38, 76, 77]. In addition, PDI levels are increased in spinal cords of sporadic ALS patients compared to nonneurological controls [38, 39]. Interestingly, PDI colocates with inclusions in motor neurons of SOD1^G93A^ mice [7], in human ALS patients [38], and also with inclusions formed by vesicle associated membrane protein-associated protein B (VAPB) in a *Drosophila melanogaster *model of a rare familial form of ALS caused by mutant VAPB [78]. These findings suggest that PDI is both upregulated in disease and recruited to areas of the cell containing aggregated protein. Furthermore, increased levels of PDI are detected in the cerebrospinal fluid (CSF) of ALS patients compared to nonneurological controls, suggesting that the level of PDI in CSF could be used as a potential biomarker of disease [38].

These studies indicate an important function for PDI in protection against mutant protein aggregation in ALS. This is supported by the increase in inclusion formation observed when mutant SOD1 expressing motor neuron-like NSC-34 cells were treated with the broad disulphide isomerase inhibitor bacitracin [7]. More recently, siRNA-mediated knockdown of PDI was also shown to increase mutant SOD1 inclusion formation in neuroblastoma cells, confirming the importance of PDI in modulating mutant SOD1 aggregation [8]. Furthermore, overexpression of PDI in neuroblastoma cells decreased the levels of insoluble mutant SOD1, inhibited inclusion formation and decreased apoptotic cell death [8]. Interestingly, a small molecular mimic of the PDI active site also decreased mutant SOD1 aggregation and inclusion formation, indicating that similar molecules may be beneficial for treatment of disease [8]. Further investigation of the effects of PDI mimics in animal models of ALS is therefore warranted.

The findings of upregulation of PDI in disease and the converse finding that overexpression is protective against mutant SOD1 in cell culture raised the question of why the increased levels of PDI in ALS were not beneficial. Recently, posttranslational modification of PDI by S-nitrosylation of critical active site cysteine residues, leading to inhibition of PDI enzymatic activity, was identified in Parkinson's and Alzheimer's disease brain tissues [59]. This same process of S-nitrosylation of PDI has now been confirmed to occur in spinal cord tissues of sporadic ALS patients as well as in transgenic SOD1^G93A^ mice, and could explain a loss of protection by PDI in disease [8]. S-nitrosylation involves the covalent addition of nitrogen monoxide (NO) to thiol side chains of cysteine residues of proteins, which can influence many cellular processes by altering both protein function and protein-protein interactions [79]. S-nitrosylation can occur in a specific substrate-dependent manner, when one or a few potential cysteine residues become modified. Nitrosative stress caused by the accumulation of reactive nitrogen species (RNS) such as peroxynitrite can cause aberrant S-nitrosylation [80]. Nitrosative stress is linked with excessive glutamate receptor activation, excitotoxicity and oxidative stress [80, 81], processes which are key events in neurodegenerative diseases including ALS [82]. Recently, the toxicity of mutant SOD1 in neuroblastoma cells has been linked with increased levels of nitric oxide [83]. 

In addition to the findings of S-nitrosylation of protein cysteine residues in ALS, a dramatic increase in tyrosine-nitrated proteins in the insoluble fractions of spinal cords from both SOD1^G93A^ mice and ALS patients was identified in a recent proteomic screen [84]. PDI was one of the nitrated proteins increased in the insoluble protein fraction from SOD1^G93A^ mice, suggesting that nitrosative stress could also lead to tyrosine nitration of PDI in ALS [84]. Previously, the PDI family member ERp57 was found to be nitrated on tyrosine residues in SOD1^G93A^ mice [85].

Importantly, a specific mechanism for subcellular redistribution of PDI has recently been identified in ALS [86, 87]. Several different members of the reticulon family of integral ER membrane proteins were shown to modulate PDI distribution and reticulon overexpression caused a change in localisation of PDI from a normal ER distribution to a less homogenous punctate pattern [86]. In SOD1^G93A^ mice, deletion of the gene encoding the reticulon-4A,B proteins accelerated disease processes, possibly by preventing the reticulon-mediated PDI redistribution [86]. The exact mechanism of the selective redistribution of PDI by reticulons remains to be determined, and how this mechanism is protective in disease remains unknown, although it is possible that redistribution allows selective interaction with a subset of PDI target proteins [87]. Although the levels of PDI are increased in the cerebrospinal fluid of transgenic SOD1^G93A^ rats and ALS patients compared to controls [38], a further unanswered question is whether the levels of PDI in other locations, such as on the cell surface or in the cytoplasm, are also affected in disease. Overall, these findings indicate that several different processes, including posttranslational modifications and subcellular redistribution, are involved in modifying PDI function in ALS, with potential implications for disease pathogenesis.

## 9. Conclusion

The recent identification of ER stress as a central process involved in ALS, and the particular involvement of PDI as a protective factor in disease, highlights new areas of research which could have potential therapeutic application. Both ER stress and PDI S-nitrosylation occur not only in mutant SOD1-linked disease, but also in the more common sporadic forms, indicating that targeting these pathways could be useful in all forms of ALS. Additionally, pharmacological prevention of protein misfolding, stimulation of autophagy or inhibition of oxidative stress could also decrease ER stress, and may be beneficial in disease. Possible applications of this research include development of small molecule PDI mimics or ER stress inhibitors, which could prove beneficial in treating this devastating disease.

## References

[1] Rothstein JD (2009). Current hypotheses for the underlying biology of amyotrophic lateral sclerosis. *Annals of Neurology*.

[2] Mitchell JD, Borasio GD (2007). Amyotrophic lateral sclerosis. *The Lancet*.

[3] Bruijn LI, Miller TM, Cleveland DW (2004). Unraveling the mechanisms involved in motor neuron degeneration in ALS. *Annual Review of Neuroscience*.

[4] Dion PA, Daoud H, Rouleau GA (2009). Genetics of motor neuron disorders: new insights into pathogenic mechanisms. *Nature Reviews Genetics*.

[5] Rosen DR (1993). Mutations in Cu/Zn superoxide dismutase gene are associated with familial amyotrophic lateral sclerosi. *Nature*.

[6] Lagier-Tourenne C, Cleveland DW (2009). Rethinking ALS: the FUS about TDP-43. *Cell*.

[7] Atkin JD, Farg MA, Turner BJ (2006). Induction of the unfolded protein response in familial amyotrophic lateral sclerosis and association of protein-disulfide isomerase with superoxide dismutase 1. *Journal of Biological Chemistry*.

[8] Walker AK, Farg MA, Bye CR, McLean CA, Horne MK, Atkin JD (2010). Protein disulphide isomerase protects against protein aggregation and is S-nitrosylated in amyotrophic lateral sclerosis. *Brain*.

[9] Schröder M (2008). Endoplasmic reticulum stress responses. *Cellular and Molecular Life Sciences*.

[10] Hayashi T, Su TP (2007). Sigma-1 receptor chaperones at the ER- mitochondrion interface regulate Ca(2+) signaling and cell survival. *Cell*.

[11] Anderson DJ, Hetzer MW (2008). Reshaping of the endoplasmic reticulum limits the rate for nuclear envelope formation. *Journal of Cell Biology*.

[12] Wu J, Kaufman RJ (2006). From acute ER stress to physiological roles of the unfolded protein response. *Cell Death and Differentiation*.

[13] Ron D, Walter P (2007). Signal integration in the endoplasmic reticulum unfolded protein response. *Nature Reviews Molecular Cell Biology*.

[14] Rutkowski DT, Kaufman RJ (2004). A trip to the ER: coping with stress. *Trends in Cell Biology*.

[15] Jonikas MC, Collins SR, Denic V (2009). Comprehensive characterization of genes required for protein folding in the endoplasmic reticulum. *Science*.

[16] Short DM, Heron ID, Birse-Archbold JLA, Kerr LE, Sharkey J, McCulloch J (2007). Apoptosis induced by staurosporine alters chaperone and endoplasmic reticulum proteins: identification by quantitative proteomics. *Proteomics*.

[17] Urano F, Wang X, Bertolotti A (2000). Coupling of stress in the ER to activation of JNK protein kinases by transmembrane protein kinase IRE1. *Science*.

[18] Ma K, Vattem KM, Wek RC (2002). Dimerization and release of molecular chaperone inhibition facilitate activation of eukaryotic initiation factor-2 kinase in response to endoplasmic reticulum stress. *Journal of Biological Chemistry*.

[19] Liu CY, Xu Z, Kaufman RJ (2003). Structure and intermolecular interactions of the luminal dimerization domain of human IRE1*α*. *Journal of Biological Chemistry*.

[20] Shen J, Snapp EL, Lippincott-Schwartz J, Prywes R (2005). Stable binding of ATF6 to BiP in the endoplasmic reticulum stress response. *Molecular and Cellular Biology*.

[21] Bernales S, Papa FR, Walter P (2006). Intracellular signaling by the unfolded protein response. *Annual Review of Cell and Developmental Biology*.

[22] Yoshida H, Matsui T, Yamamoto A, Okada T, Mori K (2001). XBP1 mRNA is induced by ATF6 and spliced by IRE1 in response to ER stress to produce a highly active transcription factor. *Cell*.

[23] Calfon M, Zeng H, Urano F (2002). IRE1 couples endoplasmic reticulum load to secretory capacity by processing the XBP-1 mRNA. *Nature*.

[24] Rao RV, Niazi K, Mollahan P (2006). Coupling endoplasmic reticulum stress to the cell-death program: a novel HSP90-independent role for the small chaperone protein p23. *Cell Death and Differentiation*.

[25] Breckenridge DG, Germain M, Mathai JP, Nguyen M, Shore GC (2003). Regulation of apoptosis by endoplasmic reticulum pathways. *Oncogene*.

[26] Kim I, Xu W, Reed JC (2008). Cell death and endoplasmic reticulum stress: disease relevance and therapeutic opportunities. *Nature Reviews Drug Discovery*.

[27] Nakayama H, Hamada M, Fujikake N (2008). ER stress is the initial response to polyglutamine toxicity in PC12 cells. *Biochemical and Biophysical Research Communications*.

[28] Duennwald ML, Lindquist S (2008). Impaired ERAD and ER stress are early and specific events in polyglutamine toxicity. *Genes and Development*.

[29] Lindl KA, Akay C, Wang Y, White MG, Jordan-Sciutto KL (2007). Expression of the endoplasmic reticulum stress response marker, BiP, in the central nervous system of HIV-positive individuals. *Neuropathology and Applied Neurobiology*.

[30] Unterberger U, Höftberger R, Gelpi E, Flicker H, Budka H, Voigtländer T (2006). Endoplasmic reticulum stress features are prominent in Alzheimer disease but not in prion diseases in vivo. *Journal of Neuropathology and Experimental Neurology*.

[31] Hoozemans JJM, van Haastert ES, Eikelenboom P, de Vos RAI, Rozemuller JM, Scheper W (2007). Activation of the unfolded protein response in Parkinson’s disease. *Biochemical and Biophysical Research Communications*.

[32] Smith WW, Jiang H, Pei Z (2005). Endoplasmic reticulum stress and mitochondrial cell death pathways mediate A53T mutant alpha-synuclein-induced toxicity. *Human Molecular Genetics*.

[33] Hetz C, Russelakis-Carneiro M, Wälchli S (2005). The disulfide isomerase Grp58 is a protective factor against prion neurotoxicity. *Journal of Neuroscience*.

[34] Ilieva EV, Naudí A, Kichev A, Ferrer I, Pamplona R, Portero-Otín M (2010). Depletion of oxidative and endoplasmic reticulum stress regulators in Pick disease. *Free Radical Biology and Medicine*.

[35] Naidoo N, Ferber M, Master M, Zhu Y, Pack AI (2008). Aging impairs the unfolded protein response to sleep deprivation and leads to proapoptotic signaling. *Journal of Neuroscience*.

[36] Turner BJ, Talbot K (2008). Transgenics, toxicity and therapeutics in rodent models of mutant SOD1-mediated familial ALS. *Progress in Neurobiology*.

[37] Saxena S, Cabuy E, Caroni P (2009). A role for motoneuron subtype-selective ER stress in disease manifestations of FALS mice. *Nature Neuroscience*.

[38] Atkin JD, Farg MA, Walker AK, McLean C, Tomas D, Horne MK (2008). Endoplasmic reticulum stress and induction of the unfolded protein response in human sporadic amyotrophic lateral sclerosis. *Neurobiology of Disease*.

[39] Ilieva EV, Ayala V, Jové M (2007). Oxidative and endoplasmic reticulum stress interplay in sporadic amyotrophic lateral sclerosis. *Brain*.

[40] Nishitoh H, Kadowaki H, Nagai A (2008). ALS-linked mutant SOD1 induces ER stress- and ASK1-dependent motor neuron death by targeting Derlin-1. *Genes and Development*.

[41] Kieran D, Woods I, Villunger A, Strasser A, Prehn JHM (2007). Deletion of the BH3-only protein puma protects motoneurons from ER stress-induced apoptosis and delays motoneuron loss in ALS mice. *Proceedings of the National Academy of Sciences of the United States of America*.

[42] Hetz C, Thielen P, Matus S (2009). XBP-1 deficiency in the nervous system protects against amyotrophic lateral sclerosis by increasing autophagy. *Genes and Development*.

[43] Ellgaard L, Ruddock LW (2005). The human protein disulphide isomerase family: substrate interactions and functional properties. *EMBO Reports*.

[44] Appenzeller-Herzog C, Ellgaard L (2008). The human PDI family: versatility packed into a single fold. *Biochimica et Biophysica Acta*.

[45] Hatahet F, Ruddock LW (2007). Substrate recognition by the protein disulfide isomerases. *FEBS Journal*.

[46] Klappa P, Ruddock LW, Darby NJ, Freedman RB (1998). The b’ domain provides the principal peptide-binding site of protein disulfide isomerase but all domains contribute to binding of misfolded proteins. *EMBO Journal*.

[47] Tian G, Xiang S, Noiva R, Lennarz WJ, Schindelin H (2006). The crystal structure of yeast protein disulfide isomerase suggests cooperativity between its active sites. *Cell*.

[48] Jessop CE, Watkins RH, Simmons JJ, Tasab M, Bulleid NJ (2009). Protein disulphide isomerase family members show distinct substrate specificity: P5 is targeted to BiP client proteins. *Journal of Cell Science*.

[49] Maattanen P, Kozlov G, Gehring K, Thomas DY (2006). ERp57 and PDI: multifunctional protein disulfide isomerases with similar domain architectures but differing substrate-partner associations. *Biochemistry and Cell Biology*.

[50] Elliott JG, Oliver JD, High S (1997). The thiol-dependent reductase ERp57 interacts specifically with N- glycosylated integral membrane proteins. *Journal of Biological Chemistry*.

[51] Garbi N, Tanaka S, Momburg F, Hämmerling GJ (2006). Impaired assembly of the major histocompatibility complex class I peptide-loading complex in mice deficient in the oxidoreductase ERp57. *Nature Immunology*.

[52] Hosoda A, Tokuda M, Akai R, Kohno K, Iwawaki T (2010). Positive contribution of ERdj5/JPDI to endoplasmic reticulum protein quality control in the salivary gland. *Biochemical Journal*.

[53] Park SW, Zhen G, Verhaeghe C (2009). The protein disulfide isomerase AGR2 is essential for production of intestinal mucus. *Proceedings of the National Academy of Sciences of the United States of America*.

[54] LaMantia M, Lennarz WJ (1993). The essential function of yeast protein disulfide isomerase does not reside in its isomerase activity. *Cell*.

[55] Lee SO, Cho K, Cho S, Kim I, Oh C, Ahn K (2010). Protein disulphide isomerase is required for signal peptide peptidase-mediated protein degradation. *EMBO Journal*.

[56] Forster ML, Sivick K, Park YN, Arvan P, Lencer WI, Tsai B (2006). Protein disulfide isomerase-like proteins play opposing roles during retrotranslocation. *Journal of Cell Biology*.

[57] Moore P, Bernardi KM, Tsai B (2010). The ero1*α*-PDI redox cycle regulates retro-translocation of cholera toxin. *Molecular Biology of the Cell*.

[58] Delom F, Mallet B, Carayon P, Lejeune PJ (2001). Role of extracellular molecular chaperones in the folding of oxidized proteins. Refolding of colloidal thyroglobulin by protein disulfide isomerase and immunoglobulin heavy chain-binding protein. *Journal of Biological Chemistry*.

[59] Uehara T, Nakamura T, Yao D (2006). S-Nitrosylated protein-disulphide isomerase links protein misfolding to neurodegeneration. *Nature*.

[60] Cheng H, Wang L, Wang CC (2010). Domain a’ of protein disulfide isomerase plays key role in inhibiting *α*-synuclein fibril formation. *Cell Stress and Chaperones*.

[61] Shashidharan P, Sandu D, Potla U (2005). Transgenic mouse model of early-onset DYT1 dystonia. *Human Molecular Genetics*.

[62] Honjo Y, Ito H, Horibe T, Takahashi R, Kawakami K (2010). Protein disulfide isomerase-immunopositive inclusions in patients with Alzheimer disease. *Brain Research*.

[63] Tanaka S, Uehara T, Nomura Y (2000). Up-regulation of protein-disulfide isomerase in response to hypoxia/brain ischemia and its protective effect against apoptotic cell death. *Journal of Biological Chemistry*.

[64] Severino A, Campioni M, Straino S (2007). Identification of protein disulfide isomerase as a cardiomyocyte survival factor in ischemic cardiomyopathy. *Journal of the American College of Cardiology*.

[65] Granell S, Baldini G, Mohammad S (2008). Sequestration of mutated *α*1-antitrypsin into inclusion bodies is a cell-protective mechanism to maintain endoplasmic reticulum function. *Molecular Biology of the Cell*.

[66] Pariser HP, Zhang J, Hausman RE (2000). The cell adhesion molecule retina cognin is a cell surface protein disulfide isomerase that uses disulfide exchange activity to modulate cell adhesion. *Experimental Cell Research*.

[67] Gilbert J, Ou WU, Silver J, Benjamin T (2006). Downregulation of protein disulfide isomerase inhibits infection by the mouse polyomavirus. *Journal of Virology*.

[68] Cho J, Furie BC, Coughlin SR, Furie B (2008). A critical role for extracellular protein disulfide isomerase during thrombus formation in mice. *Journal of Clinical Investigation*.

[69] Curbo S, Gaudin R, Carlsten M (2009). Regulation of interleukin-4 signaling by extracellular reduction of intramolecular disulfides. *Biochemical and Biophysical Research Communications*.

[70] Turano C, Coppari S, Altieri F, Ferraro A (2002). Proteins of the PDI family: unpredicted non-ER locations and functions. *Journal of Cellular Physiology*.

[71] Ramachandran N, Root P, Jiang XM, Hogg PJ, Mutus B (2001). Mechanism of transfer of NO from extracellular S-nitrosothiols into the cytosol by cell-surface protein disulfide isomerase. *Proceedings of the National Academy of Sciences of the United States of America*.

[72] Wilkinson B, Gilbert HF (2004). Protein disulfide isomerase. *Biochimica et Biophysica Acta*.

[73] Cheng HJ, Lei HY, Lin CF (2009). Anti-dengue virus nonstructural protein 1 antibodies recognize protein disulfide isomerase on platelets and inhibit platelet aggregation. *Molecular Immunology*.

[74] Watts JC, Huo H, Bai Y (2009). Interactome analyses identify ties of PrP and its mammalian paralogs to oligomannosidic N-glycans and endoplasmic reticulum-derived chaperones. *PLoS pathogens*.

[75] Tabata Y, Takano K, Ito T (2007). Vaticanol B, a resveratrol tetramer, regulates endoplasmic reticulum stress and inflammation. *American Journal of Physiology*.

[76] Massignan T, Casoni F, Basso M (2007). Proteomic analysis of spinal cord of presymptomatic amyotrophic lateral sclerosis G93A SOD1 mouse. *Biochemical and Biophysical Research Communications*.

[77] Ahtoniemi T, Jaronen M, Keksa-Goldsteine V, Goldsteins G, Koistinaho J (2008). Mutant SOD1 from spinal cord of G93A rats is destabilized and binds to inner mitochondrial membrane. *Neurobiology of Disease*.

[78] Tsuda H, Han SM, Yang Y (2008). The amyotrophic lateral sclerosis 8 protein VAPB is cleaved, secreted, and acts as a ligand for Eph receptors. *Cell*.

[79] Hess DT, Matsumoto A, Kim SO, Marshall HE, Stamler JS (2005). Protein S-nitrosylation: purview and parameters. *Nature Reviews Molecular Cell Biology*.

[80] Nakamura T, Lipton SA (2010). Preventing Ca2+-mediated nitrosative stress in neurodegenerative diseases: possible pharmacological strategies. *Cell Calcium*.

[81] De Palma C, Falcone S, Panzeri C, Radice S, Bassi MT, Clementi E (2008). Endothelial nitric oxide synthase overexpression by neuronal cells in neurodegeneration: a link between inflammation and neuroprotection. *Journal of Neurochemistry*.

[82] Uehara T (2007). Accumulation of misfolded protein through nitrosative stress linked to neurodegenerative disorders. *Antioxidants and Redox Signaling*.

[83] Arciello M, Capo CR, Cozzolino M (2010). Inactivation of cytochrome c oxidase by mutant SOD1s in mouse motoneuronal NSC-34 cells is independent from copper availability but is because of nitric oxide. *Journal of Neurochemistry*.

[84] Basso M, Samengo G, Nardo G (2009). Characterization of detergent-insoluble proteins in ALS indicates a causal link between nitrative stress and aggregation in pathogenesis. *PloS ONE*.

[85] Casoni F, Basso M, Massignan T (2005). Protein nitration in a mouse model of familial amyotrophic lateral sclerosis: possible multifunctional role in the pathogenesis. *Journal of Biological Chemistry*.

[86] Yang YS, Harel NY, Strittmatter SM (2009). Reticulon-4A (Nogo-A) redistributes protein disulfide isomerase to protect mice from SOD1-dependent amyotrophic lateral sclerosis. *Journal of Neuroscience*.

[87] Walker AK (2010). Protein disulfide isomerase and the endoplasmic reticulum in amyotrophic lateral sclerosis. *Journal of Neuroscience*.

